# Baicalein Long-Circulating Liposomes Improve Histological and Functional Outcomes After Traumatic Brain Injury

**DOI:** 10.1155/adpp/2869332

**Published:** 2024-11-22

**Authors:** Dong Yuan, Wenbo Xu, Faisal Raza, Hajra Zafar, Shuangxian Guan, Zhen Wang, Kamran Hidayat Ullah, Hongchao Shi

**Affiliations:** ^1^Department of Emergency, JinTan Hospital Affiliated to JiangSu University, 500 Jintan Dadao, Jintan, Changzhou 213200, Jiangsu, China; ^2^School of Pharmacy, Shanghai Jiao Tong University, Shanghai 200240, China; ^3^Department of Pharmacy, CECOS University of IT and Emerging Sciences, Peshawar, Pakistan; ^4^Department of Critical Care Medicine, Shanghai General Hospital, Shanghai Jiao Tong University School of Medicine, 85 Wujin Road, Shanghai 200080, China

**Keywords:** baicalein, liposomes, long-circulating, traumatic brain injury

## Abstract

High mortality and disability have been principally linked with traumatic brain injury (TBI) with this condition being a principal issue in public health. Currently, there is no reliable pharmaceutical agent to salvage the damage caused by TBI. Baicalein (BCL), a traditional Chinese medicine active ingredient, has preliminarily shown repair activity on brain injury. However, BCL has poor water solubility and bioavailability, which culminates in rapid elimination in vivo. Herein, we sought to solve the above-mentioned challenges that are associated with the application of this flavonoid by preparing BCL-loaded long-circulating liposomes (BLC-Lips) via thin-film hydration method. Subsequently, BLC-Lips were applied to TBI model mice to evaluate their effect on brain injury repair. The results showed that the prepared BLC-Lips exhibited smaller sized nanoparticles, excellent polydispersed index (PDI), and zeta (*ζ*)-potential with stable property. After loading of BCL into the liposomes, we estimated the physicochemical properties of BLC-Lips to be roughly 87.98% (encapsulation efficiency [EE]) and 7.56% (loading capacity of the drug). Administration of BLC-Lips through oral route increased aqueous solubility, bioavailability, and time for in vivo circulation of BCL. Moreover, the BLC-Lips could improve outcomes of histological and neurological motor function and reduce inflammatory cytokines and neurotoxicity after TBI. Taken together, the long-circulating liposomes may serve as a new approach to potentially prolong drug circulation in vivo and increased bioavailability of BCL for TBI treatment.

## 1. Introduction

Globally, traumatic brain injury (TBI) is considered as a major issue in public health in view of its high morbidity and mortality worldwide [1]. Regarding pathophysiology of the condition, TBI occurs when the function of the brain changes or when external force induces brain lesions as indicated by other evidence [2]. Annually, TBI affects over 50 million individuals worldwide [3], while roughly an estimated half of the populations in the world are likely to develop one or more lifetime TBIs [4]. Epilepsy, cognitive impairment and dementia, Parkinson's disease, and stroke have been discovered as the potential long-run risk of TBI [5]. However, there is still no reliable pharmaceutical agent to salvage the damage caused by TBI even after broad research on brain trauma including medical and surgical intervention [6]. The enormous burden and societal costs of TBI make it the need of the hour to address.

The root of *Scutellaria baicalensis* is a natural source of essential flavonoids like baicalein (BCL). Numerous beneficial effects of BCL include antiviral, antioxidation, anti-inflammation, and cardiovascular protection [7]. Through experimental Parkinson's disease and cerebral ischemia, scientists have established the neuroprotective properties of BCL [8]. Meanwhile, existing study has shown that BCL can improve histological and functional outcomes after TBI [9]. Unfortunately, various factors contribute to poor bioavailability of BCL, namely, low aqueous solvability, quick intestinal breakdown, increased metabolism, and quick elimination of this flavonoid from the bloodstream [10]. Extensive searches of the literature have indicated that some nanocarriers have been developed to solve the aforementioned problems of BCL. Noted among them are BCL-loaded nanostructured lipid carriers and BCL nanocrystals [11, 12]. Since this research is in the preliminary stage, there is the need to develop more nanocarriers for wider applications of BCL. Therefore, we sought to increase the solubility of BCL by developing liposome for increased treatment efficacy.

Structurally, liposomes contain hydrophobic tail and hydrophilic head wherein upon contact with inorganic and organic solvents can form globular vesicles [13]. Through compatibility principles, hydrophobically vesicles can easily incorporate lipophilic drugs. Also, there is distribution of negative charges on liposomal vesicles' outer surfaces [14]. Of note, this provides a bridge in which the hydrophilic materials interact with lipophilic drugs. Although BCL has many beneficial effects, its hydrophobicity coupled with low bioavailability makes it difficult to attain its pharmacological potential, and hence, preparation into liposome may be best approach. This is because liposomes, especially those prepared with long-circulating materials, may potentially improve stability, prolong circulation times, and increase the bioavailability of BCL. Modification of long-circulating liposomes with poly(ethylene glycol) (PEG) has been described to prolong the circulation time of drugs in bloodstream with rapid elimination of drugs by reticuloendothelial system (RES) [15] being prevented. Until now, little is known about the potential treatment efficacy of BCL-loaded long-circulating liposomes (BLC-Lips) for TBI application.

In this study, we prepared PEG-modified BLC-Lips with thin-film hydration method [16]. After optimization of the formulation, we further characterized it in vitro, prior to in vivo evaluation. The effects of BLC-Lips in TBI mice model were ascertained, while the possible mechanism of BLC-Lips on TBI was also explored. Putatively, BLC-Lips could be an effective treatment to attenuate brain injury in TBI animals.

## 2. Materials and Methods

### 2.1. Materials

Macklin (Shanghai, China) provided BCL (purity ≥ 98%), wogonoside, and lecithin. The cholesterol and methoxy-polyethylene-glycol-di-stearoyl-phosphatidylethanolamine (DSPE-PEG2000) were supplied by Aladdin (Shanghai, China). TEDIA (Ohio, USA) provided chromatography-grade methanol (high purity), while Sinopharm (Shanghai, China) supplied the remaining chemical reagents.

### 2.2. Determination of BCL Concentration With High-Performance Liquid Chromatographic (HPLC) Technique

Using C18 column (5 *μ*m; 250 × 4.6 mm, Waters, Ireland) of HPLC (Agilent, USA), we determined BCL concentration in vitro. The following conditions were set up for the analytical procedure, namely, 30°C as temperature of column, 280 nm as wavelength for detection, aqueous solution of 0.1% phosphoric acid, and methanol (45:55, v: v) at flowing at a rate of 1.0 mL/min as the mobile phase and injection volume of 20 *μ*L. After HPLC analysis of BCL, we constructed standard curve for the estimation of BCL concentration, wherein the curve was linear (*Y* = 126.32 × *C* + 2.4133) at a range of 0.1–50 *μ*g/mL with the coefficient of determination of *R*^2^ = 0.9975. Notably, BCL concentration was denoted as C, while peak areas were represented as *Y*. Also, in vivo BCL concentration was similarly determined as mentioned above with internal standard being wogonoside (50 *μ*g/mL). The above-mentioned HPLC conditions were employed to successfully separate BCL and wogonoside from endogenous substances. Later, we constructed standard curves for the determination of BCL concentration in vivo, wherein *Y* = 0.0175 × *C* + 0.0063 (liner range: 0.05–20 *μ*g/mL, *R*^2^ = 0.9976).

### 2.3. Preparation of BLC-Lips

Preparation of BLC-Lips was accomplished with thin-film hydration method [17]. The long-circulating liposomes composed of lecithin, cholesterol, and DSPE-PEG2000. Ultrasonication was employed to dissolve appropriate amount of BCL, lecithin, cholesterol, and DSPE-PEG2000 in absolute ethanol (10 mL). Subsequently, we removed the ethanol with rotovap operating under appropriate temperature and pressure. Afterward, the thin lipid film that was formed at round-bottom flask was dried for 4 h. Ultimately, we hydrated the film with sterile water. Prior to further experiments, the prepared BLC-Lips were stored at 4°C.

### 2.4. Optimization of BLC-Lips Preparation

Orthogonal design (L9 (3)^4^) of experiment via MINITAB 16 software was utilized to optimize BLC-Lips preparation. Variables that were selected for the optimization process were (A) lecithin/cholesterol (w/w) ratio, (B) lecithin/BCL (w/w) ratio, (C) lecithin/DSPE-PEG2000 (w/w) ratio, and (D) blank (Table 1). The success of the optimization process was evaluated with the size of particles as the evaluation indicator.

### 2.5. Physical Characterization of BLC-Lips

#### 2.5.1. Size, Polydispersed Index (PDI), and Zeta (*ζ*)-Potential

Measurement of size, PDI, and *ζ*-potential of the liposomal particles was accomplished with NanoBrook-90Plus PALS package (Brookhaven, USA). Before determination, the BLC-Lips suspensions were diluted 10 times with sterile water. At conditions of 25°C temperature and 90° angle of scattering, we placed the samples in cuvettes and determined the above-mentioned characteristics.

#### 2.5.2. Morphological Observation of BLC-Lips With Transmission Electron Microscopic (TEM) Technique

The TEM (JEM-200CX, Jeol, Japan) technique was applied to observe the morphology of BLC-Lips. Prior to morphological visualization with TEM, we negatively stained (30 s) the diluted BLC-Lips with a solution of phosphotungstate (2%) on a copper support grid. Later, we dried the stained BLC-Lips for 30 min with an infrared lamp.

#### 2.5.3. Estimation of Entrapment Efficiency (EE) and Loading Capacity of BCL

Ultracentrifugation method was utilized to calculate EE and loading capacity of BCL that has been incorporated in BLC-Lips [18]. The BLC-Lips suspension was put in ultrafiltration tubes before 10 min of centrifugation at 4°C and speed of 10,000 g. Later, we collected the supernatant and analyzed via HPLC system, in which it was recorded as the quantity of BCL in BLC-Lips. Afterward, we calculated the EE% and loading capacity of BCL (DL%) with the equations as follows:

(1) $$\begin{array}{c} \text{EE}\%=\left(\frac{\text{Total quantity of BCL in BCL}-\text{Lips}-\text{free BCL}}{\text{Total quantity of BCL in BCL}-\text{Lips}}\right)\times 100,\\ \text{DL}\%=\left(\frac{\text{Total quantity of BCL in BCL}-\text{Lips}-\text{free BCL}}{\text{Total }q\text{uantity of BCL}-\text{Lips}}\right)\times 100. \end{array}$$

#### 2.5.4. Testing of BLC-Lips Stability

To test for the stability of BLC-Lips during storage, we evaluated changes in physical characteristics such as size, PDI, loading capacity of BCL, and EE at 4°C and 25°C. Prior to the evaluation of the above-mentioned attributes, we stored BLC-Lips for distinct period of time (0, 7, and 14 days). Also, we stored BLC-Lips in phosphate-buffered saline (PBS) (pH 7.4) to investigate the stability of the liposome based on existing literature [19].

### 2.6. Testing of In Vitro BCL Release From Liposomes

The release of BCL in vitro from liposomes was tested using the dialysis method [20]. Dialysis membrane (MV 3000 Da) was socked in boiling water overnight before used. Using BCL and BLC-Lips, we tested for liposomal effect on drug release in vitro in 3 distinct dissolution media, namely, pH 7.4 PBS, pH7.0 sterile water, and pH 1.2 hydrochloric acid (HCl). Later, we filled the membrane bags equally with BCL or BLC-Lips in equivalent drug content and agitated in a shaker at 100 rpm. The release medium (2 mL) was collected at the indicated intervals (0.08, 0.25, 0.5, 0.75, 1, 1.5, 2, 4, 6, 8, 12, 24, and 36 h), while a fresh medium was replaced at the same volume. Afterward, we analyzed BCL content in the sampled media with HPLC analysis. The cumulative release (CR%) of BCL release from BLC-Lips was calculated as ratio to total BCL used in liposomal formulation with the following equations:

(2) $$\begin{array}{c} \text{CR}\%=\left(\frac{\text{cumulative amount of BCL in the release medium}}{\text{total amount of BCL in BCL}-\text{Lips}}\right)\times 100\%. \end{array}$$

Also, we investigated the release mechanism of BCL and BLC-Lips before fitting the data to different release models (like zero-order, first-order, Higuchi, and Ritger–Peppas) as described in existing literature with some modifications [21]. KinetDS 2.0 software was utilized to perform nonlinear least squares regression and estimate *R*-squared (*R*^2^), wherein models with higher values of *R*^2^ were considered as better adjusting the data set [22, 23].

### 2.7. Pharmacokinetic and Biodistribution of BLC-Lips In Vivo

Sprague Dawley rats (male, 180–220 g) and C57BL/6 mice (male, 18–22 g) for the following in vivo experiments were supplied and approved (certification number: SCXK (su)2023–0017) by Jiangsu University Institute for Animal Care and Use Committee. We cared for and handled the animals in accordance with guidelines issued by the National Institutes of Health. Based on an earlier work [24], we gave the same dose (200 mg/kg) to the mice (*n* = 6) in BCL and BLC-Lips groups through intragastric route. Sampling of blood (0.5 mL) from the rats (postorbital venous plexus) was performed at predesigned time intervals (0.125, 0.25, 0.5, 0.75, 1, 1.5, 2, 3, 4, 6, 8, 10, 12, 24, 36, 48, and 72 h), after which the blood was collected into sodium heparin containing Eppendorf tubes. Plasma was obtained after 10 min of centrifugation of sampled blood at 4°C and 3700 rpm. We mixed plasma, 50 *μ*L of wogonoside (50 *μ*g/mL, internal standard), and 750 *μ*L of ethyl acetate to extract BCL. After vortexing for 1 min and allowing to stand for a while, we removed the ethyl acetate layer via decantation. The two extractions were combined, and the ethyl acetate layer was dried with N_2_ at 37°C. Afterward, aliquot (400 *μ*L) of mobile phase solution was added prior to 10 min of centrifugation (10,000 rpm). Analysis of the supernatants was carried out according to the above-mentioned chromatographic condition. We recorded the peak areas of BCL and wogonoside, before calculating drug concentrations of BCL through substitution into the in vivo standard curve. Subsequently, BAPP software (Nanjing, China, was applied to calculate the main variables of pharmacokinetics, namely as peak times, maximal concentration (*C*_max_) of BCL, time for BCL to attain *C*_max_ (*T*_max_), elimination half-life of BCL (*T*_1/2_), average time for BCL to reside in the rat's body (MRT), and plasma BCL concentration area under the curve (AUC_0∼∞_) via noncompartmental analysis.

In accordance with previous work [25], we allotted the mice (40) into two groups, namely, BCL and BLC-Lips groups. After fasting the mice for 2 h and giving them unrestricted access to water, we intragastrically administered BCL and BLC-Lips (200 mg/kg) to them. Later, we appropriately collected tissues (like heart, liver, spleen, lung, and brain) from the mice at 2, 1.5, 0.75, and 0.25 h. Normal saline was used to rinse the tissues before diluting with PBS (5 times) concordance with weight and subsequent homogenization and centrifugation (10 min, 12,298 g). Aliquot (200 *μ*L) supernatant was treated as indicated earlier in this section before at −80°C and construction of a standard curve to determine concentration of BCL in the heart, liver, spleen, lung, and brain based on results of pre-experiments (Table 1).

### 2.8. Effects of BLC-Lips on TBI Mice

#### 2.8.1. Establishment of TBI Mice Model

Construction of TBI mouse model was performed based on previous work [26]. Prior to carrying out this experiment, we subjected the C57BL/6 mice to sham injury or injury. Sodium pentobarbital (50 mg/kg, i.p.) was utilized to anesthetize the mice before they were placed on a stereotaxic device. We created bone window over the right skull at a diameter of 5 mm and left the dura intact after we incised the scalp along the median. Later, we freely dropped a 25-g metal bolt from 40-cm height using a brain trauma percussion device, which resulted in unilateral cortical contusion on the right hemisphere. Subsequently, we anesthetized the sham-injured mice, but they were not concussively injured. For the mice to regain consciousness, we placed them in ambient air.

#### 2.8.2. Treatment of TBI Mice With BLC-Lips

Randomization of mice that underwent TBI into 8 groups (*n* = 6) was carried out. Based on preliminary trials and existing literature [27], the mice were allotted into sham, model, low-dose BCL (B-L, 50 mg/kg), medium-dose BCL (B-M, 100 mg/kg), high-dose BCL (B-H, 200 mg/kg), low-dose BLC-Lips (BLC-Lips-L, 50 mg/kg), medium-dose BLC-Lips (BLC-Lips-M, 100 mg/kg), and high-dose BLC-Lips (BLC-Lips-H, 200 mg/kg) groups. A day after TBI establishment in mice, we administered each mouse with the above-mentioned dosage forms via intragastric route for seven consecutive days. Animals in sham and model groups were administered intragastrically with physiological saline (0.9%).

#### 2.8.3. Neurological Behavior Assessment

After TBI mice have received the aforementioned dosage forms for 1 week, we evaluated level of neurological function with modified neurological severity scores (mNSS). Element of mNSS involves tests such as balance, reflex, sensory, and motor. Higher score reflects more severe injury, wherein the scores were as follows: Mild injury ranged 1–6, moderate injury ranged 7–12, and severe injury ranged 13–18. Consecutively, we conducted the experiment once a day for 7 days.

Assessment of recovery of motor function of TBI mice was performed with forelimb asymmetry functional test. We placed the mice in a translucent cylinder (which had a diameter of 20 cm and height of 30 cm), wherein they tended to spontaneously use their forelimbs to touch the cylinder wall. Later, we recorded the number of mice that had contralateral (affected), ipsilateral (unaffected), and bilateral limb contact with the wall for 3–10 min. Scoring of behavior was carried out according to existing criteria [28].

#### 2.8.4. Assessment of Brain Water Content (BWC)

Detection of the status of brain edema was performed with BWC [29]. In each group, we selected 3 mice that were decapitated before taking out their brains. Later, we determined the wet weight of the brains, before drying them for 24 h in an oven at 60°C. Afterward, we determined the dry weight and calculated the BWC using the following formula:

(3) $$\begin{array}{c} \text{BWC}\left(\%\right)=\left(\frac{\text{Wet weight }–\text{ Dry weight}}{\text{Wet weight}}\right)\times 100\%. \end{array}$$

#### 2.8.5. Assaying of Blood–Brain Barrier (BBB) Permeability

Quantification of changes in BBB permeability was performed with Evans blue infiltration method [30]. After 72 h of injury, we injected the mice with a solution (4 mL/kg, 2%) of Evans blue via the femoral vein. The mouth, nose, and limbs of the mice turned blue through blood circulation. After 2 h of cycling, we anesthetized the mice and perfused them with heparin sodium saline (200 mL, 0.9% NaCl, and 20 U/mL heparin) at 50 mL/min through the left atrium. Brain tissues were dissected and weighed, before they were placed in N,N-dimethylformamide solution (1 mL/100 mg), homogenized, and incubated at 60°C for 24 h. Later on, we extracted the supernatant via centrifugation (10,000 g, 25 min) and subsequent measurement of absorbance (OD) value (OD value) with spectrophotometric technique at 630 nm. The OD values of Evans blue with different gradients were measured, and the standard curves were plotted. The Evans blue content of samples was calculated from the standard curve.

#### 2.8.6. Inflammatory Factors and Neurotoxicity Assays

After sacrificing the mice, we collected the blood and dissected the brains. Subsequently, the brain tissue was lysed into protein solution by radioimmune precipitation assay (RIPA), wherein we centrifugated blood before extraction of supernatant as serum. Through enzyme-linked immunosorbent assay (ELISA) kits, we detected inflammatory cytokines (interleukin [IL]-1*β* and IL-18) and neurotoxicity (lactate dehydrogenase [LDH]) of brain tissues and serums.

#### 2.8.7. Hematoxylin and Eosin (HE) Staining

The HE staining was utilized to analyze the brain lesions. Prior to capturing images of HE-stained tissue with an upright microscope (Nikon, Japan), we carried out the following procedure. We performed staining with HE after 24 h of postfixation of brains coupled with embedment of sections (5 *μ*m) with paraffin.

#### 2.8.8. Data Analysis

Triplicate measurements of experimental data were carried out before we expressed as means ± standard deviation (SD). A Student's *t*-test was used for comparisons between two groups, while one-way analysis of variance (ANOVA) was applied for comparisons among multiple groups. GraphPad Prism 8.0 (USA) was applied in this study for statistical analysis. In terms of significant levels, *p* < 0.05,  *p* < 0.01, and *p* < 0.001 were considered.

## 3. Results and Discussion

### 3.1. Determination of BCL Concentration via HPLC Analysis

**Figure 1:**
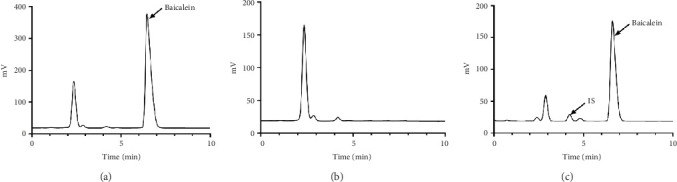
High-performance liquid chromatographic (HPLC) analysis in vitro and in vivo. (a) Baicalein (BCL); (b) blank plasma; (c) blank plasma mixed with BCL and internal standard (wogonoside).

Figure 1(a) depicts HPLC chromatogram of BCL determination in vitro. The peak of BCL was well separated with 6.8 min as the retention time. Figures 1(b) and 1(c) display HPLC chromatogram of the blank plasma, wogonoside, and BCL in vivo. From the figures, we observed that the peaks of BCL and wogonoside were good, albeit absence of endogenous impurities' peaks. Besides, we discovered a good separation of both peaks of both peaks with the respective retention times of BCL and wogonoside were 6.8 and 4.2 min. Based on the above results, we selected wogonoside as suitable for the internal standard. Consequently, the established method was feasible for detecting BCL in vitro and in vivo.

### 3.2. Preparation of BLC-Lips

Preliminary, we investigated BLC-Lips formulation by determining factors that affect the preparation of the aforementioned liposomes via the orthogonal design of experiment. As shown in Table 2, the order in which the three factors affect the size of BLC-Lips particles was as follows: *C* > *B* > *A*. Inferably, the radio of lecithin to DSPE-PEG2000 > the radio of lecithin to BCL > the ratio of lecithin to cholesterol. The best mass ratio of lecithin to cholesterol was screened to be 6:1. Notably, the optimal levels of lecithin to BCL and lecithin to DSPE-PEG2000 ratios were both 8:1. Therefore, the optimal condition for the preparation of BLC-Lips was as follows: BCL (5 mg), lecithin (40 mg), cholesterol (6.66 mg), and DSPE-PEG2000 (5 mg). Liposomes with long-circulating characteristics were modified with DSPE-PEG2000 [31]. A previous study has shown that liposomes coated with DSPE-PEG2000 could enhance oral bioavailability of a hydrophobic drug [32]. Therefore, our selected optimal condition is appropriate for the preparation of BCL-loaded long-circulating liposomes. Also, thin-film hydration method (also known as Bangham method) was employed to develop BLC-Lips because it is regarded as the simplest and commonest technique and has been utilized for the preparation of all types of liposomes. This method was chosen for the preparation of BLC-Lips because of the following advantages of the technique [16, 33]:

(i) Regarding preparation of liposomes, this technique is simple to perform and has been accepted widely. (ii) In this method, common solvents of organic nature (such as methanol, chloroform, etc.) are used to dissolve lipids. (iii) The method can produce homogeneously unilamellar vesicles after the hydrated lipid phase has been extruded. (iv) The method can produce liposomes that are loaded with single and dual drugs via passive loading technique. Leveraging the advantages of this technique, most scientists have employed thin-film hydration method to produce different types of liposomes.

### 3.3. Characterization of BLC-Lips

**Figure 2:**
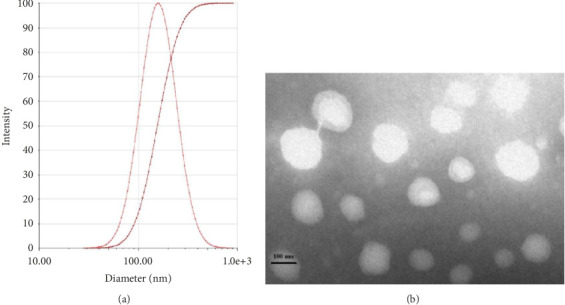
Characterization of baicalein long-circulating liposomes (BLC-Lips). (a) The particle size distribution of BLC-Lips; (b) the morphology (transmission electron microscopic (TEM) images) of BLC-Lips.

Herein, characterization of BLC-Lips was carried out using physical attributes such as size of particles, *ζ*-potential, PDI, morphology, EE, and DL under study and stability. Consequently, Figure 2 shows the results of size distribution and morphology of BLC-Lips, while Table 3 summarizes the detailed stability study.

BLC-Lips showed single- and narrow-sized particles' distribution (Figure 2(a)) with mean size of BLC-Lips particles being 158.37 ± 2.68 nm and PDI of 0.186 ± 0.006. In addition, the prepared BLC-Lips exhibited higher EE and DL of 87.85 ± 1.43% and 7.55 ± 0.17%, respectively. As important variables in elimination of nanocarriers, sizes of nanoparticle greatly influence their circulation and biological distributions of loaded drugs [34]. Hence, prolonged in vivo circulation of nanoloaded drugs with sizes < 200 nm may potentially circumvent RES-induced rapid elimination [35]. Relatively, lower PDI value may also imply increased stability of nanoparticles [36], which was confirmed in subsequent stability studies. The *ζ*-potential for BLC-Lips was −28.19 ± 0.36 mV. Existing literature has indicated that the presence of negative charges on nanoparticles can facilitate the formation of monodispersed droplets, which may improve BLC-Lips stability [37]. The relatively high absolute *ζ*-potential (above 20 mV) values indicate a strong capability to limit the possibility of conglutination and thus promote maintenance of nanodrug homogeneity [38]. The morphology of BLC-Lips revealed that BLC-Lips were monodispersed with about 100 nm as the particle size (Figure 2(b)). Also, TEM images displayed similar particle diameter with what was measured via Particle Sizer.

To affirm that the increased stability of BLC-Lips with lower values of PDI, we assessed storage stability for Days 7 and 14 at 4°C and 25°C in DDW and PBS buffer (Table 3). During the storage stage, we did not observe any turbidity and sediment formed upon visual examination. Similarly, we did not neither discover any substantial changes (*p* > 0.05) in size of liposomal particles, PDI, loading capacity of BCL, and EE after Days 7 and 14, nor at 4°C or 25°C (Table 3) in the two media. The remarkable stability of BLC-Lips might be attributed to the core–shell structure which thus provided more particle stability [39].

### 3.4. BCL Release In Vitro From BLC-Lips

**Figure 3:**
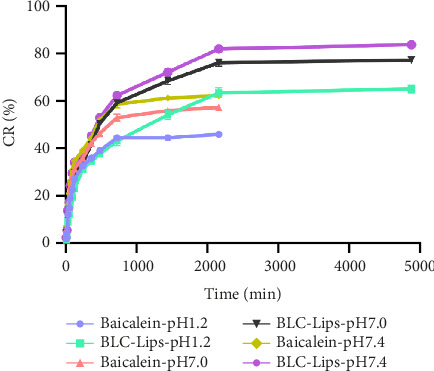
The in vitro drug release behaviors of the free baicalein (BCL) and BCL long-circulating liposomes (BLC-Lips) in hydrochloric acid (HCl, pH 1.2), water (pH 7.0), and phosphate-buffered saline (PBS, pH 7.4).

Figure 3 displays the pattern of in vitro release of BCL and BLC-Lips in the aforementioned 3 dissolution media. It was found that BLC-Lips cumulatively release an increased amount of BCL in the media compared to unloaded BCL. In this regard, smaller sized BLC-Lips may have caused the observed trend of drug release, which suggests the potential of nanoliposomes to dissolve BCL and substantially increase simulated release of the drug. Additionally, the order of accumulative release of BCL and BLC-Lips in different media was as follows: PBS > water > HCl, wherein this trend may be attributable to the solubility of BCL being dependent on pH. This observation suggests that pH could affect drug release. Meanwhile, compared with BCL, the BLC-Lips demonstrated an apparently sustained and slow release. It has been suggested that lipophilic drugs mostly release from liposomal nanocarriers under the influence of hydrophobic interaction between liposomal core and the drug. Thus, for drugs to slowly release from liposomes, the interactions of drugs with polymer chains in liposomal core should be strongly enough [40]. Besides, BCL released slowly from liposomes in HCl condition, which suggests the possibility of liposomes withstanding simulated intestinal fluid (pH 1.2), probably because they can incorporate drugs stably in their inner cores coupled with the potential to induce firm and prolonged release of drugs.

To limit the deviations of theoretical values from practical values, we fitted the data that were obtained from the in vitro release of BCL from BLC-Lips to the zero-order, first-order, Higuchi, and Ritger–Peppas models (Table 4) [41]. Based on the best fitted model, we selected the most appropriate model for the drug release using *R*^2^ [42].

The data for release kinetics of BCL from BLC-Lips showed that the models that better fitted the data in the various media were first-order (DDW, *R*^2^ = 0.943; HCl, *R*^2^ = 0.935; PBS, *R*^2^ = 0.923) and Ritger–Peppas (DDW, *R*^2^ = 0.910; HCl, *R*^2^ = 0.937; PBS, *R*^2^ = 0.927). The mechanism of BCL release from BLC-Lips may suggest anomalous (non-Fickian) diffusion. This observation suggests that the release of BCL from BLC-Lips occurs combination of factors including sustained mechanism, but not only via diffusion driven by high concentrations as stated by the law of Fick [43, 44]. The results of other studies corroborate this finding [45, 46].

### 3.5. Pharmacokinetic and Biodistribution Analysis of BLC-Lips

**Figure 4:**
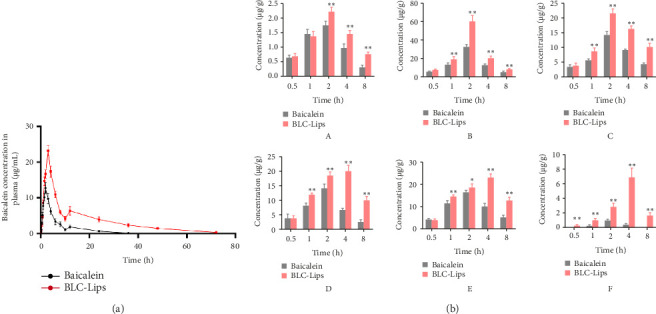
Pharmacokinetic and biodistribution of BLC-Lips in vivo. (a) Plasma concentration–time profiles of free baicalein and baicalein long-circulating liposomes (BLC-Lips); (b) the drug concentrations in various tissues of rats at different time points (A: heart; B: liver; C: spleen; D: lung; E: kidney; F: brain; ⁣^∗^*p* < 0.05 vs. baicalein group; ⁣^∗∗^*p* < 0.01 vs. baicalein group).

The pharmacokinetic parameters of free BCL and BLC-Lips in rats after intragastrical administration are illustrated in Table 5.  Figure 4(a) displays pattern of plasma concentration versus time curve for free BCL and BLC-Lips. In previous studies [47], BCL has shown poor absorption coupled with quick clearance from circulation, which result in rapid *T*_1/2_ and poor in vivo availability. Therefore, we developed BCL-loaded long-circulating liposomes to mainly delay clearance of BCL but increase its in vivo availability. Compared to free BCL, the BLC-Lips increased the *C*_max_ dramatically (23.22 ± 1.48 *μ*g/mL vs. 12.76 ± 1.44 *μ*g/mL, *p* < 0.001). At each point, we also observed increased BCL concentration in plasma of rats that received BLC-Lips compared to free BCL. Thus, concentration of BCL released from BLC-Lips increased substantially. With regard to BCL clearance, we could still detect the flavone up to 72 h for BLC-Lips, but at 36 h BCL from the free BCL group could not be noticed. Moreover, substantial prolonged *T*_1/2_ (20.31 ± 1.84 min vs. 7.23 ± 0.44 min, *p* < 0.001) may suggest that BLC-Lips could delay BCL elimination and MRT (19.68 ± 1.28 min vs. 8.48 ± 0.47 min, *p* < 0.001). More importantly, the AUC values of BLC-Lips were almost 4 times that of the free BCL. Through these observations, it is possible to conclude that long-circulating liposomes prolonged retention of BCL in vivo, which might have increased bioavailability of the drug, and thus corroborated sustained in vitro release result. In general, different types of liposomes have been observed to demonstrate a release pattern of biphasic nature, which involves rapid release of the drug from liposomes at the initial stage before controlled release [48].

Within about 20 min, we discovered that BCL was rapidly released from BLC-Lips at the initial stage, which may be owing to BCL that had not been encapsulated into liposomes and controlled release of entrapped BCL [49]. Virtually, the BLC-Lips displayed a controlled release of BCL between roughly 15–70 h, but that of BCL concentration in plasma diminished around 35 h, which may be ascribed to potential convention of BCL to baicalin, a derivative of BCL [50]. The release pattern of BCL agrees with existing literature [51, 52].

Existing literature has reported that pegylated liposomes have reduced surface–surface interactions including their interactions with plasma proteins [53]. Besides, Chiu and colleagues posited that DSPE-PEG 2000 (15 mol %) could effectively protect phosphatidylserine-based liposomes against high affinity and binding plasma proteins. Since understanding the interactions between liposomes and plasma proteins is vital for the optimization of liposomal design, safety, and efficacy, we intend to comprehensively investigate the interactions of BLC-Lips with plasma proteins and their effect on stability, circulation time, and ability of the liposomes to target specific tissues or cells in our not-too-distant future experiments [54].

As shown in Figure 4(b), the drug content (*μ*g/g) in various tissues of the two groups of rats showed a trend of first increasing and then decreasing. At 2–4 h, the drug concentration in the various tissues of rats in the BCL and BLC-Lips groups reached the highest value with the drugs mainly distributed in liver, kidney, lung, and other tissues and then showed varying degrees of decrease. In addition, after 1 h, the drug content in various tissues of BLC-Lips group rats was higher than that of the BCL group. Especially in the BLC-Lips group, the drug content in the brain significantly increased with statistical significance (*p* < 0.01). This indicates that BLC-Lips could significantly improve the absorption and distribution of BLC in mice, especially by increasing its content in the brain.

### 3.6. Effect of BLC-Lips on TBI In Vivo

**Figure 5:**
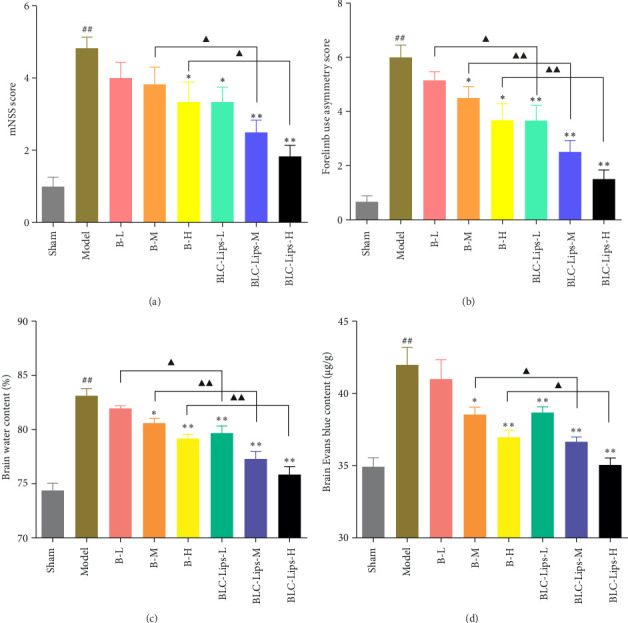
Neurological and motor function and brain recovery outcomes. (a) Modified neurological severity scores (mNSS) scores; (b) forelimb use asymmetry scores; (c) brain water content; (d) brain Evans blue content (*p* < 0.01 vs. sham group; ⁣^∗^*p* < 0.05 vs. model group; ⁣^∗∗^*p* < 0.01 vs. model group; ^▲^*p* < 0.05 vs. same dose of baicalein group; ^▲▲^*p* < 0.01 vs. same dose of baicalein group).

To assess neurological and motor function, we performed the mNSS and forelimb asymmetry tests in TBI animal model. It can be seen in Figure 5(a) that mNSS scores in the model group were obviously different from the sham batch (*p* < 0.01). Also, we observed a significant decreased mNSS scores of mice in B-H (*p* < 0.05), BLC-Lips-L (*p* < 0.05), BLC-Lips-M (*p* < 0.01), and BLC-Lips-H (*p* < 0.01) groups compared to the model group. Meanwhile, the mNSS scores of mice in BLC-Lips-M and BLC-Lips-H groups were lower than those in the groups that received same dose of BCL (*p* < 0.05). In the forelimb asymmetry test, we observed similar results in terms of the mNSS scores across the treated groups (Figure 5(b)). Similarly, we observed an obvious decline of forelimb use asymmetry scores in B-M (*p<*0.05), B-H (*p* < 0.05), BLC-Lips-L (*p* < 0.01), BLC-Lips-M (*p* < 0.01), and BLC-Lips-H (*p* < 0.01) groups compared to the model group. Meanwhile, the forelimb uses asymmetric scores of mice in BLC-Lips-L (*p* < 0.05), BLC-Lips-M (*p* < 0.01), and BLC-Lips-H (*p* < 0.01 groups were significantly lower than those in the groups that received same dose of BCL. These results showed that deficits in neurological and motor function were significantly attenuated after treatment with BCL and BLC-Lips, whereas BLC-Lips showed a better therapeutic effect.

The BBB leakage and brain edema were detected in TBI mice after the administration of different doses of BCL and BLC-Lips. Brain Evans blue concentration in the administration group reduced significantly compared to the TBI model group. Besides, we observed a substantial increase in BWC of TBI mice compared to sham batch. At the same time, the treatment group showed marked decrease in BWC and concentration of Evans blue (Figures 5(c) and 5(d)), which contributed to the recovery of extravasation due to BBB dysfunction. Earlier works have suggested that prognosis after TBI could be improved through the maintenance of BBB integrity [55, 56]. It is possible that liposomes without ligand coating may passively target the brain during TBI via various alternative mechanisms that can increase drug accumulation in the brain without binding to a particular ligand–receptor. Scientists have shown that distribution of liposome-based drug to the brain depends on the properties of a particular drug and characteristics of the liposomal formulation [57]. Boyd and colleagues discovered that an enhanced permeability and retention (EPR) like effect facilitated opening of the BBB to stealth liposomes during TBI, which increased delivery of drugs to injury site of the brain [58]. More importantly, materials like DSPE-PEG2000 polymer can be used to control the size and liposomal release properties, which can prolong the release of BCL and increase its accumulation in the brain injury site. In this regard, Hu and coexperimenters developed DSPE-PEG2000 polymer liposome which prolonged the release of temozolomide and quercetin as well as enhanced their accumulation in the brain [59]. Therefore, we leveraged on this existing literature to develop BLC-Lips that can passively target BCL to the brain. Nevertheless, future research will consider coating the surface of BLC-Lips with brain-targeting ligands like aquaporin-4, anti-VCAM-1, C4-3 aptamer, etc., which have been found to enhance biodistribution of drugs in the brain [60].

Also, we confirmed that the treatment of TBI animals with BCL and BLC-Lips could attenuate BBB dysfunction and decreased brain edema, all of which may be attributed to reduced inflammatory [61] activities of BCL and BLC-Lips. Subsequently, we therefore validated these using inflammatory factor assay.

**Figure 6:**
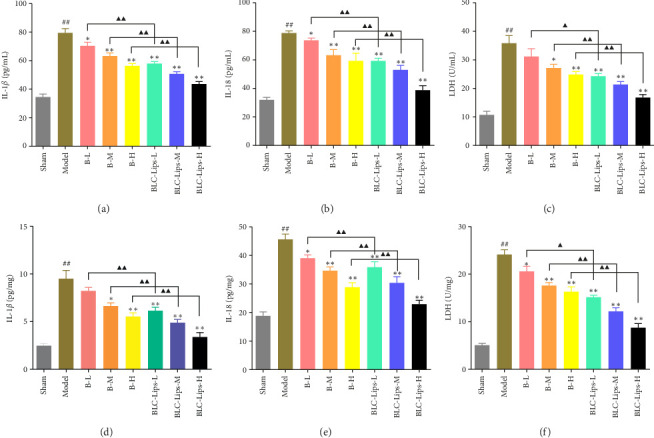
The inflammatory factors (interleukin [IL]-1 beta (*β*) and IL-18) and neurotoxicity (lactate dehydrogenase (LDH) assay. (a) IL-1*β* in serum; (b) IL-18 in serum; (c) LDH in serum; (d) IL-1*β* in brain; (e) IL-18 in brain; (f) LDH in brain (*p* < 0.01 vs. sham group; ⁣^∗^*p* < 0.05 vs. model group; ⁣^∗∗^*p* < 0.01 vs. model group; ^▲^*p* < 0.05 vs. same dose of baicalein group; ^▲▲^*p* < 0.01 vs. same dose of baicalein group).

In response to TBI-induced inflammation, some common proinflammatory cytokines released were IL-1*β* and IL-18, which have been discovered to mostly cause neuron death during brain dysfunction. A type of cascade of reactions involving neurotoxicity and inflammatory response occurs when secondary brain injury after TBI ensues [62, 63]. Thus, we performed ELISA to detect IL-1*β* and IL-18. Compared to the sham group, increased inflammatory cytokine levels (IL-1*β* and IL-18) and neurotoxicity (LDH) were observed in brain serum and serum of the model group. However, IL-1*β*, IL-18, and LDH concentrations in BCL and BLC-Lips treated groups' serum and brain were reduced obviously (Figure 6), which implies decrease in inflammation and neurotoxicity. The above results suggest that BCL and BLC-Lips could relieve TBI by restraining inflammatory reaction and neurotoxicity. As a proinflammatory cytokine, IL-1*β* is released by activated microglia, which then promotes inflammation in neurons, disruption of BBB, and apoptosis of neurons, and concomitantly culminates in secondary injury [64–66]. Although it is a crucial proinflammatory cytokine in the central nervous system, IL-6 can also demonstrate anti-inflammatory activity through the protection of neurons via the regulation of immune responses and support repair of tissues [67]. Thus, chronic inflammation and degeneration of neurons occur when the activity of IL-6 is prolonged. As a biomarker of cellular injury and necrosis, increased levels of LDH suggest extensive damage to neurons and dysfunction of metabolism owing to hypoxic conditions and tissue injury in TBI [68]. Based on the available literature, it is evidently clear that IL-1*β*, IL-6, and LDH play vital roles regarding mediation of inflammation and injury to the neurons. Therefore, decrease in levels of the two cytokines and enzymes may be served as the underlying mechanism of anti-inflammatory and neuroprotective properties of BLC-Lips. Notwithstanding, the detailed molecular pathways involved in the anti-inflammatory and neuroprotective effects of BLC-Lips will be studied comprehensively in our future works.

**Figure 7:**
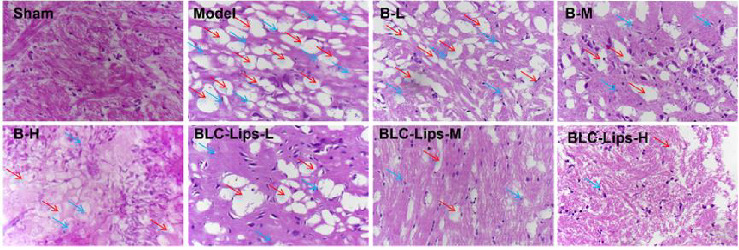
Histopathological analysis of brains with different treatments (20x). The red arrow indicates cellular edema. The blue arrow indicates that the cell nucleus has been destroyed.

The results of HE stains (Figure 7) revealed that brain tissue of the sham group had normal cortical structure, clear cell hierarchy, and neuronal nuclei as well as no hemorrhage, edema, injury, or vasodilation. After the trauma, the trauma site and its surrounding tissues became visibly edematous with slight swelling of neuronal and glial cell cytosol and visible nucleus consolidation or fragmentation. After BCL and BLC-Lips treatment, the degree of edema and the swelling of nerve cells in the traumatized area tissue decreased markedly compared to the model group. Some of the vacuolated central cells were absent, while the degree of nuclear fixation was improved. There was no obvious difference in histopathology between the BLC-Lips group and the same dose-free BCL group. These results showed that BCL and BLC-Lips could effectively improve the TBI repair ability.

## 4. Conclusions

In summary, orthogonal design was applied to optimize the formulation of BLC-Lips prior to their successful preparation via the thin-film hydration method. The prepared BLC-Lips had smaller sized particles, lower PDI, and *ζ*-potential with stable property. The EE and loading capacity of BCL were around 87.98% and 7.56%. Importantly, the BLC-Lips notably increased solvability, oral in vivo availability, and circulation time of BCL. Moreover, BLC-Lips could improve histological and neurological motor function outcomes and reduce inflammatory cytokines and neurotoxicity after TBI. Taken together, the long-circulating liposomes may serve as a new approach to potentially prolong drug circulation in vivo and increased bioavailability of BCL for TBI treatment.

## Figures and Tables

**Table 1:** Prescription composition of baicalein long-circulating liposomes (BLC-Lips) preparation.

Levels	Factors		
	(A) Lecithin: cholesterol	(B) Lecithin: baicalein	(C) Lecithin: DSPE-PEG2000
1	8:1	10:1	8:1
2	6:1	8:1	6:1
3	5:1	6:1	4:1

**Table 2:** Data obtained from orthogonal experimental design of baicalein long-circulating liposomes (BLC-Lips) formulation.

Groups	Factors				Particle size (mm)
	*A*	*B*	*C*	*D*	
1	1	1	1	1	251.38
2	1	2	2	2	276.12
3	1	3	3	3	351.87
4	2	1	2	3	311.83
5	2	2	3	1	276.98
6	2	3	1	2	177.73
7	3	1	3	2	366.62
8	3	2	1	3	218.67
9	3	3	2	1	274.61
*k*_1_	879.37	929.83	647.78	802.97	
*k*_2_	766.54	771.77	862.56	820.47	
*k*_3_	859.90	804.21	995.47	882.37	
*k*_1_/3	293.12	309.94	215.93	267.66	
*k*_2_/3	255.51	257.26	287.52	273.49	
*k*_3_/3	286.63	268.07	331.82	294.12	
*R*	37.61	52.69	115.90	26.47	

**Table 3:** Stability of baicalein long-circulating liposomes (BLC-Lips, *n* = 3, mean ± SD).

Time	Day 0	Day 7	Day 14	*p* value
4°C				
Diameter (nm)	162.67 ± 1.83	163.18 ± 2.02	163.93 ± 2.11	= 0.48
PDI	0.191 ± 0.004	0.193 ± 0.006	0.194 ± 0.008	= 0.59
DL (%)	7.58 ± 0.16	7.51 ± 0.27	7.25 ± 0.31	= 0.18
EE (%)	87.75 ± 1.28	87.12 ± 1.39	86.75 ± 1.69	= 0.46
25°C				
Diameter (nm)	161.95 ± 2.17	163.58 ± 2.29	165.63 ± 2.85	= 0.15
PDI	0.195 ± 0.009	0.195 ± 0.014	0.198 ± 0.015	= 0.78
DL (%)	7.61 ± 0.12	7.18 ± 0.21	7.13 ± 0.31	= 0.07
EE (%)	87.55 ± 1.36	86.08 ± 2.58	84.59 ± 2.12	= 0.11

*Note:p* value: Day 14 versus Day 0.

**Table 4:** The fitting results of the release curves of BLC and BLC-Lips.

Media		Model	Fitting equation	*R* ^2^
DDW	BLC	Zero-order	Mt = 0.02*x* + 22.00	0.574
		First-order	Mt = 51.67 (1 − e^−0.008t^)	0.945
		Higuchi	Mt = 1.24*x*^1/2^ + 11.36	0.818
		Ritger–Peppas	Mt = 6.53*x*^0.30^	0.910
	BLC-Lips	Zero-order	Mt = 0.01*x* + 25.86	0.550
		First-order	Mt = 72.01 (1 − e^−0.003t^)	0.943
		Higuchi	Mt = 1.22*x*^1/2^ + 12.27	0.828
		Ritger–Peppas	Mt = 6.28*x*^0.31^	0.917

HCl (pH 1.2)	BLC	Zero-order	Mt = 0.02*x* + 18.77	0.512
		First-order	Mt = 42.42 (1 − e^−0.008t^)	0.969
		Higuchi	Mt = 0.99*x*^1/2^ + 10.07	0.770
		Ritger–Peppas	Mt = 5.81*x*^0.29^	0.879
	BLC-Lips	Zero-order	Mt = 0.01*x* + 19.94	0.599
		First-order	Mt = 59.14 (1 − e^−0.003t^)	0.935
		Higuchi	Mt = 1.02*x*^1/2^ + 8.72	0.863
		Ritger–Peppas	Mt = 4.53*x*^0.33^	0.937

PBS (pH 7.4)	BLC	Zero-order	Mt = 0.02*x* + 24.61	0.570
		First-order	Mt = 56.26 (1 − e^−0.008t^)	0.935
		Higuchi	Mt = 1.34*x*^1/2^ + 13.14	0.814
		Ritger–Peppas	Mt = 7.49*x*^0.29^	0.910
	BLC-Lips	Zero-order	Mt = 0.02*x* +28.15	0.566
		First-order	Mt = 76.54 (1 − e^−0.003t^)	0.923
		Higuchi	Mt = 1.29*x*^1/2^ + 13.83	0.838
		Ritger–Peppas	Mt = 6.97*x*^0.31^	0.927

**Table 5:** Plasma pharmacokinetic parameters of baicalein long-circulating liposomes (BLC-Lips) in rats.

Parameters	Free baicalein	BLC-Lips
*C*_max_ (*μ*g/mL)	12.76 ± 1.44	23.22 ± 1.48⁣^∗∗∗^
*T*_max_ (h)	2.00	3.00
*T*_1/2_ (h)	7.23 ± 0.44	20.31 ± 1.84⁣^∗∗∗^
AUC_0-∞_ (*μ*g/mL∗h)	73.95 ± 3.77	265.24 ± 4.98⁣^∗∗∗^
MRT (h)	8.48 ± 0.47	19.68 ± 1.28⁣^∗∗∗^

⁣^∗∗∗^*p* < 0.001, significant as compared to the free baicalein group.

## Data Availability

Data will be available upon request to the corresponding author.
